# Potentially inappropriate medications according to PRISCUS list and FORTA (Fit fOR The Aged) classification in geriatric psychiatry: a cross-sectional study

**DOI:** 10.1007/s00702-022-02541-1

**Published:** 2022-09-02

**Authors:** Martin Schulze Westhoff, Adrian Groh, Sebastian Schröder, Phileas Johannes Proskynitopoulos, Kirsten Jahn, Martin Klietz, Benjamin Krichevsky, Dirk O. Stichtenoth, Felix Wedegärtner, Stefan Bleich, Helge Frieling, Johannes Heck

**Affiliations:** 1 Department of Psychiatry, Social Psychiatry and Psychotherapy, Hannover Medical School, Carl-Neuberg-Str. 1, 30625 Hannover, Germany; 2 Department of Neurology, Hannover Medical School, Hannover, Germany; 3 Medical Service of the German Armed Forces, Kiel, Germany; 4 Institute for General Practice and Palliative Care, Hannover Medical School, Hannover, Germany; 5 Institute for Clinical Pharmacology, Hannover Medical School, Hannover, Germany; 6 Drug Commissioner of Hannover Medical School, Hannover, Germany

**Keywords:** Potentially inappropriate medications, Pharmacotherapy safety, Geriatric psychiatry, FORTA, PRISCUS

## Abstract

Adverse drug reactions (ADRs) constitute a frequent cause of hospitalization in older people. The risk of ADRs is increased by the prescription of potentially inappropriate medications for older people (PIMs). The PRISCUS list and the FORTA classification represent established tools to detect PIMs. The aim of the present study was to examine the prevalence and characteristics of PIM prescriptions on the gerontopsychiatric ward of a university hospital in Germany. To this aim, medication charts of 92 patients (mean age 75.9 ± 7.7 years; 66.3% female) were analyzed on a weekly basis until patient discharge by utilization of the PRISCUS list and the FORTA classification. Overall, 335 medication reviews comprising 2363 drug prescriptions were analyzed. 3.0% of the prescribed drugs were PIMs according to the PRISCUS list, with benzodiazepines and Z-drugs accounting for nearly half (49.3%) of all PIM prescriptions. 30.4% of the patients were prescribed at least one PRISCUS-PIM, while 43.5% of the study population took at least one FORTA class D drug. A considerable proportion of gerontopsychiatric patients were affected by PIMs; however, the overall number of PIM prescriptions in the study population was low. Further improvements in the quality of prescribing should target the use of sedating agents such as benzodiazepines and Z-drugs. Physicians should be aware of discrepancies between the PRISCUS list and the FORTA classification.

**Supplementary Information:**

The online version contains supplementary material available at 10.1007/s00702-022-02541-1.

## Introduction

The risk of adverse drug reactions (ADRs) increases in older patient populations (Davies and O'Mahony 2015) due to impaired organ function, physiologically altered pharmacodynamic and pharmacokinetic characteristics, and the presence of polypharmacy (Price et al. 2014). Polypharmacy is commonly defined as the simultaneous intake of five or more different drugs (Mortazavi et al. 2016). Advanced age and polypharmacy represent the most important risk factors for the prescription of potentially inappropriate medications for elderly people (PIMs) (Stock et al. 2014). PIMs are characterized by an unfavorable benefit-to-risk ratio (e.g. due to pronounced anticholinergic side effects) and are associated with an increased probability of ADRs (Lohman et al. 2017). Gerontopsychiatric patients represent an at-risk population for the prescription of PIMs and the occurrence of ADRs (Wolff et al. 2021).

Several PIM classification systems have been developed in recent years and their use in clinical practice has been investigated extensively (Krüger et al. 2021). One of the first systems to be applied were Beers criteria, which were developed in the United States (By the 2019 American Geriatrics Society Beers Criteria^®^ Update Expert Panel 2019). In Germany, the PRISCUS list and the Fit fOR The Aged (FORTA) classification are preferred to Beers criteria as they are specifically tailored to the German pharmaceutical market (Pazan et al. 2022; Siebert et al. 2013). While the PRISCUS list has repeatedly been evaluated in the clinical setting and its importance in preventing ADRs has been assessed, the utility and importance of the FORTA classification have much less intensely been studied in practice (de Agustín Sierra et al. 2021; Schubert et al. 2013). Since psychotropic drugs represent the largest group among PIMs according to both the PRISCUS list and the FORTA classification, an increased incidence of PIM prescriptions can be assumed on gerontopsychiatric wards.

Therefore, the present study aimed at investigating the prevalence and characteristics of PIM prescriptions in geriatric psychiatry based on the PRISCUS list and the FORTA classification. In particular, our study focused on the differences between these two PIM classification systems. The basis of our cross-sectional study were weekly medication reviews conducted by an interdisciplinary expert panel on the gerontopsychiatric ward of the Department of Psychiatry, Social Psychiatry and Psychotherapy of Hannover Medical School.

## Methods

### Ethics approval

The study was approved by the Ethics Committee of Hannover Medical School (No. 10206_BO_K_2022) and adhered to the Declaration of Helsinki and its later amendments.

### Eligibility criteria

Patients were enrolled in the study (i) if they were ≥ 65 years of age, (ii) if they were treated on the gerontopsychiatric ward of the Department of Psychiatry, Social Psychiatry and Psychotherapy of Hannover Medical School between April 2021 and February 2022, and (iii) if they or their legal representative had provided written informed consent that patient-related data be used for clinical research. Hannover Medical School is a large university hospital and tertiary care referral center in northern Germany. The gerontopsychiatric ward is a 27-bed facility specialized on the treatment and care of elderly psychiatric patients.

### Data acquisition

A convenience sample of 92 patients were consecutively enrolled in the study between April 2021 and February 2022. The medication charts of the enrolled patients were reviewed on a weekly basis until patient discharge by an interdisciplinary expert panel comprising specialists in psychiatry, neurology, internal medicine, geriatrics, and clinical pharmacology. All drugs taken by the patients on a regular basis were analyzed with the aid of the PRISCUS list and the FORTA classification. Drugs taken by the patients on an as-needed basis (i.e. *pro re nata* drugs) were excluded from the analysis.

The PRISCUS list (*priscus* (Latin), ancient, venerable) tabulates 83 drugs considered as PIMs (Siebert et al. 2013). The PRISCUS list is tailored to the German pharmaceutical market, and it applies to people ≥ 65 years of age. In addition to listing PIMs, the PRISCUS list provides suggestions of suitable pharmacological alternatives for the treatment of elderly people. For the purpose of this study, we categorized the drug prescriptions in our study population as PIMs (according to the PRISCUS list), non-PIMs (i.e. drugs not listed as PIMs on the PRISCUS list), and suitable therapeutic alternatives to PIMs (according to the PRISCUS list).

The FORTA classification categorizes drugs into four classes (i.e. A to D), based on their therapeutic indications: A = indispensable drugs in the pharmacological treatment of elderly people; B = drugs with proven or obvious efficacy in elderly people; C = drugs with questionable efficacy–safety profiles in elderly people; D = drugs that should be avoided in elderly people (Pazan et al. 2022). In this study, drugs not mentioned in the FORTA classification were classified as “not labelled”. Similar to the PRISCUS list, the FORTA classification was developed in Germany, and it also applies to people ≥ 65 years of age.

Demographic characteristics—i.e. age, sex, and International Statistical Classification of Diseases and Related Health Problems 10th Revision (ICD-10) diagnoses—were retrieved from the patient records.

### Statistical analysis

Continuous variables are depicted as means ± standard deviations (SDs) or as medians with interquartile ranges (IQRs). For categorical variables, absolute and relative frequencies were calculated. All statistical analyses were performed with IBM^®^ SPSS^®^ Statistics for Windows, version 28 (Armonk, New York, USA).

## Results

### Study population, medication reviews, and drug prescriptions

The mean age of the study population (*n* = 92) was 75.9 ± 7.7 years and two thirds of the patients were female (66.3%; 61/92) (Table 1). Dementia was the most frequent psychiatric diagnosis in the study population (39.1%; 36/92), followed by depression (37.0%; 34/92) and schizophrenia or schizophreniform disorder (18.5%; 17/92). The most prevalent somatic comorbidity was arterial hypertension, which affected nearly two thirds (66.3%; 61/92) of the study population.

**Table 1:** Characteristics of the study population (*n* = 92)

Variables	*n*	%
Sex		
Female	61	66.3
Male	31	33.7
Psychiatric diagnoses^a^		
Depression^b^	34	37.0
Bipolar affective disorder^c^	6	6.5
Schizophrenia or schizophreniform disorder^d^	17	18.5
Mental and behavioral disorder due to use of alcohol, tobacco, or sedatives or hypnotics^e^	16	17.4
Dementia^f^	36	39.1
Delirium^g^	15	16.3
Other psychiatric disorder(s)	9	9.8
Somatic diagnoses^a^		
Arterial hypertension	61	66.3
Coronary heart disease	15	16.3
Chronic heart failure	10	10.9
Atrial fibrillation	21	22.8
Status post stroke	9	9.8
Type-2 diabetes mellitus	13	14.1
Chronic obstructive pulmonary disease	6	6.5
Hypothyroidism	13	14.1
Urinary tract infection	7	7.6
Other somatic disorder(s)	85	92.4

The mean age ± standard deviation of the study population was 75.9 ± 7.7 years

*ICD-10* International Statistical Classification of Diseases and Related Health Problems 10th Revision

^a^Patients could have more than one diagnosis

^b^ICD-10 F32, F33

^c^ICD-10 F31

^d^ICD-10 F06.2, F2X

^e^ICD-10 F10, F13, F17

^f^ICD-10 F00, F01, F02, F03

^g^ICD-10 F05

Overall, 335 medication reviews were conducted during the study period, with a median of 3 medication reviews per patient (IQR 2–5; range 1–18 medication reviews per patient). A total of 2363 drugs were prescribed in the study population, representing 182 individual agents (Supplementary Table 1). The three most frequently prescribed drugs were risperidone (5.0%; 119/2363), ramipril (4.0%; 94/2363), and tinzaparin (3.8%; 90/2363). On average (mean ± SD), 7.1 ± 4.1 drugs were analyzed per medication review (one medication review corresponding to one patient).

### Potentially inappropriate medications for older people according to the PRISCUS list

**Fig. 1:**
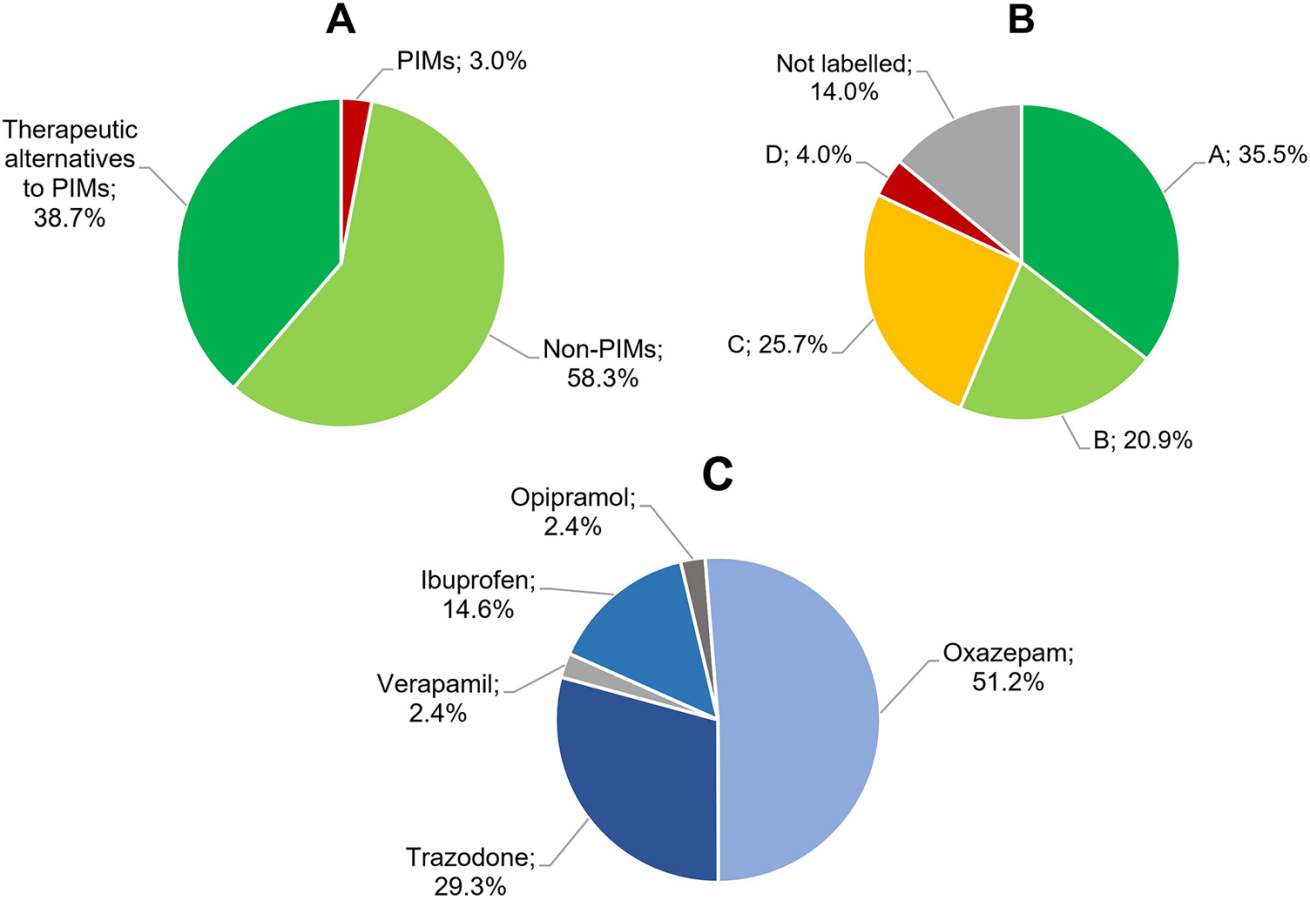
**A**–**C** Categorization of drug prescriptions (*n* = 2363) in the study population according to the PRISCUS list (**A**) and the FORTA classification (**B**). Prescriptions (*n* = 41) of drugs that are indicated as possible therapeutic alternatives to PIMs in the PRISCUS list, but that are contradictorily labeled as class D drugs according to the FORTA classification, are shown in (**C**). PIM denotes potentially inappropriate medication for elderly people (i.e. ≥ 65 years of age), FORTA Fit fOR The Aged. FORTA classes A to D are defined as follows: A = indispensable drugs in the pharmacological treatment of elderly people; B = drugs with proven or obvious efficacy in elderly people; C = drugs with questionable efficacy–safety profiles in elderly people; and D = drugs that should be avoided in elderly people

3.0% of all prescribed drugs (71/2363) were PIMs according to the PRISCUS list (Fig. 1A) and 30.4% (28/92) of all patients received at least one PRISCUS-PIM. The three most frequently prescribed PIMs were lorazepam > 2 mg/d (23.9%; 17/71), clozapine (14.1%; 10/71), and olanzapine > 10 mg/d (9.9%; 7/71) (Table 2). Taken together, benzodiazepines and Z-drugs accounted for nearly half of all PIM prescriptions (49.3%; 35/71) in the study population.

**Table 2:** Absolute and relative frequencies of potentially inappropriate medications for older people according to the PRISCUS list that were detected in the study population

PIM	*n*	%
All PIMs	71	100
Lorazepam > 2 mg/day	17	23.9
Clozapine	10	14.1
Olanzapine > 10 mg/day	7	9.9
Alprazolam	6	8.5
Diazepam	6	8.5
Zopiclone > 3.75 mg/day	6	8.5
Digoxin	5	7.0
Doxazosin	5	7.0
Fluoxetine	4	5.6
Beta-acetyldigoxin	3	4.2
Etoricoxib	2	2.8

*PIM* potentially inappropriate medication for older people (i.e. ≥ 65 years of age)

### Categorization of drug prescriptions according to the FORTA classification

Of all drugs prescribed in the study population, 35.5% (838/2363), 20.9% (493/2363), 25.7% (607/2363), and 4.0% (94/2363) were categorized as FORTA class A, B, C, and D drugs, respectively (Fig. 1B). Remarkably, 43.5% (40/92) of the study population took at least one FORTA class D drug, while 93.5% took at least one FORTA class C drug (86/92). Of note, 14.0% (331/2,363) of the prescribed drugs were not mentioned in the FORTA classification system (category “Not labelled” in Fig. 1B).

Risperidone (19.6%; 119/607), pipamperone (11.0%; 67/607), and mirtazapine (9.2%; 56/607) constituted the three most frequently prescribed FORTA class C drugs, while oxazepam (22.3%; 21/94), aripiprazole (12.8%; 12/94), and trazodone (12.8%; 12/94) represented the three most frequently prescribed FORTA class D drugs (Table 3).

**Table 3:** Absolute and relative frequencies of FORTA class C drugs (i.e. drugs with questionable efficacy–safety profiles in elderly people) and FORTA class D drugs (i.e. drugs that should be avoided in elderly people) prescribed in the study population

Drug	*n*	%
FORTA class C drugs	607	100
Risperidone	119	19.6
Pipamperone	67	11.0
Mirtazapine	56	9.2
Lorazepam	50	8.2
Quetiapine	48	7.9
Venlafaxine	48	7.9
Bisoprolol	45	7.4
Spironolactone	36	5.9
Olanzapine	25	4.1
Melperone	19	3.1
Bupropion	13	2.1
Pregabalin	12	2.0
Valproic acid	10	1.6
Zopiclone	10	1.6
Duloxetine	6	1.0
Digoxin	5	0.8
Doxazosin	5	0.8
Fluoxetine	4	0.7
Naloxone	4	0.7
Nebivolol	4	0.7
Beta-Acetyldigoxin	3	0.5
Digitoxin	3	0.5
Tianeptine	3	0.5
Oxycodone	2	0.3
Phenprocoumon	2	0.3
Tilidine	2	0.3
Tramadol	2	0.3
Levofloxacin	1	0.2
Metoprolol	1	0.2
Morphine	1	0.2
Trospium chloride	1	0.2
FORTA class D drugs	94	100
Oxazepam	21	22.3
Aripiprazole	12	12.8
Trazodone	12	12.8
Clozapine	10	10.6
Diclofenac	7	7.4
Alprazolam	6	6.4
Diazepam	6	6.4
Ibuprofen	6	6.4
Agomelatine	5	5.3
Haloperidol	4	4.3
Etoricoxib	2	2.1
Ciprofloxacin	1	1.1
Opipramol	1	1.1
Verapamil	1	1.1

*FORTA* Fit fOR The Aged

### Discrepancies between the PRISCUS list and the FORTA classification

Interestingly, 41 drug prescriptions in the study population referred to agents that were indicated as suitable therapeutic alternatives to PIMs in the PRISCUS list, while at the same time being labeled as class D drugs according to the FORTA classification (Fig. 1C). Oxazepam (51.2%; 21/41) was the most frequently prescribed of these agents with contradictory evaluations by the PRISCUS list and the FORTA classification, followed by trazodone (29.3%; 12/41) and ibuprofen (14.6%; 6/41). Conversely, there were no drug prescriptions in the study population of agents designated as PIMs according to the PRISCUS list while simultaneously being labeled as FORTA class A drugs.

## Discussion

The present study investigated the prevalence and characteristics of PIM prescriptions on the gerontopsychiatric ward of a university hospital in Germany over a period of approximately ten months. The analysis was based on weekly medication reviews conducted by an interdisciplinary expert panel comprising specialists from psychiatry, neurology, internal medicine, geriatrics, and clinical pharmacology. Two different PIM classification systems, i.e. the PRISCUS list and the FORTA classification were utilized.

The study population displayed high similarities to prior studies (Moebs et al. 2020; Seifert et al. 2022) in terms of age, sex, and comorbidity profiles. The mean age of the study population was approximately 76 years and the three most prevalent psychiatric diagnoses were dementia, depression, and schizophrenia/schizophreniform disorders.

The frequency and characteristics of PIM prescriptions in the elderly general population have been studied extensively in the past (de Agustín Sierra et al. 2021; Lohman et al. 2017; Price et al. 2014), and it has consistently been demonstrated that a significant proportion of geriatric patients receive PIMs (de Agustín Sierra et al. 2021; Lohman et al. 2017). The proportion of patients affected by PIM prescriptions varied substantially between 20 and 60% (de Agustín Sierra et al. 2021; Lohman et al. 2017), presumably owing to different study designs and settings. In the context of geriatric psychiatry, there are also several studies that have investigated the frequency and risk factors for PIM prescriptions (Hefner et al. 2021; Moebs et al. 2020; Seifert et al. 2022). Hefner and colleagues reported that 33.9% of geriatric psychiatric patients in a multicenter, retrospective analysis received a PRISCUS-PIM (Hefner et al. 2021), albeit without validation of the results by another PIM classification system such as the FORTA classification. In the literature, risk factors for PIM prescriptions include polypharmacy and a diagnosis of schizophrenia, whereas dementia appears to be more of a protective factor (Davies and O'Mahony 2015; Price et al. 2014). This could explain why the prevalences of PIM prescriptions in collectives of patients with dementia, with proportions of 14–22%, were comparatively lower than in other studies with more heterogeneous study populations (Cross et al. 2016; Fiss et al. 2013; Wucherer et al. 2017).

In our study, we detected that approximately 30% of all patients were prescribed a PRISCUS-PIM, whereas 43.5% received a FORTA class D drug. These data are overall consistent with results from Moebs et al., who described a proportion of 41% of all patients as PIM-positive based on Beers criteria in a comparable gerontopsychiatric patient cohort (Moebs et al. 2020). To date, only a few studies have examined the proportion of PIM prescriptions in relation to all medication prescriptions in the respective study populations. Seifert et al. reported that 5.7% of all drug prescriptions on the gerontopsychiatric wards of a hospital were PIMs according to the PRISCUS list (Seifert et al. 2022). This is overall in agreement with the results of the present study, where we identified a proportion of 3% of all prescribed drugs as PRISCUS-PIMs. In contrast, previous studies did not examine the proportion of drugs of questionable benefit or the proportion of drugs that should be avoided altogether according to the FORTA classification in the gerontopsychiatric context. To date, only data from Greten and co-workers in geriatric patients with Parkinson’s disease (PD) exist (Greten et al. 2021). With regard to non-antiparkinson medications (which included psychotropic drugs such as antidepressants and antipsychotics), Greten et al. found that 40.9% and 26.9% of the agents were FORTA class A or B drugs, respectively (Greten et al. 2021). By contrast, 17.7% of the drugs were problematic according to the FORTA classification (13.9% class C drugs; 3.8% class D drugs). In the present study, we identified a similar prescribing pattern in geriatric psychiatry: a total of 29.7% of the prescribed agents were problematic according to FORTA (25.7% class C drugs; 4.0% class D drugs).

The results of our study suggest that a substantial proportion of drugs prescribed in geriatric psychiatry should at least be discussed critically according to the FORTA classification (i.e. FORTA class C drugs), while the proportion of agents to be avoided (i.e. FORTA class D drugs) is similar to the proportion of PRISCUS-PIMs (4.0% and 3.0%, respectively). This observation can be explained by the fact that the FORTA classification, which features recommendations (graded from A to D) for 296 drugs, is significantly more comprehensive and refined compared to the PRISCUS list with only 83 listed drugs considered unsuitable for older people (Pazan et al. 2020; Siebert et al. 2013). However, it must be taken into consideration that both the PRISCUS list and the FORTA classification have not been specifically designed for the use in geriatric psychiatry, but for elderly patients in general. Therefore, a rational assessment of the medications prescribed in geriatric psychiatry requires thorough benefit–risk analyses as well as equally careful evaluations of possible pharmacological alternatives. As a general rule in medicine, the first priority must be not to harm the patient (i.e. the principle of non-maleficence (*primum non nocere*), as laid down in the Hippocratic Oath). As part of diligent benefit–risk analyses it must also be considered what the harm to the patient is if medication is *not* taken. In this regard, the clinical significance of psychiatric disorders with respect to quality of life, the prognosis of comorbid somatic illnesses, as well as the risk of suicidality need to be taken into account by healthcare professionals who treat elderly patients suffering from both mental and physical disorders. Unfortunately, it is not always possible that only the medication with the fewest side effects can be used. However, the medication with the fewest side effects should be used first, and during the further course of drug treatment it must be constantly re-evaluated whether the potential benefit or the potential harm of the medication outweighs.

The most commonly prescribed PRISCUS-PIMs in our study population were lorazepam > 2 mg/day, clozapine, and olanzapine > 10 mg/day. This finding is largely in accordance with other studies that reported benzodiazepines (lorazepam > 2 mg/day, diazepam) but also antipsychotics (haloperidol > 2 mg/day, olanzapine > 10 mg/day) as the most frequently prescribed PIMs (Hefner et al. 2021; Seifert et al. 2022). In addition, the use of doxepin and amitriptyline was also noted (Moebs et al. 2020; Seifert et al. 2022).

In the present study, the most commonly prescribed FORTA class C drugs were risperidone, pipamperone, and mirtazapine, while oxazepam, aripiprazole, and trazodone represented the most commonly prescribed FORTA class D agents. In a collective of geriatric PD patients, clozapine and oxazepam were the two most common FORTA class D drugs (of all prescribed non-antiparkinson drugs) (Greten et al. 2021).

The use of sedating agents is a highly debated subject in geriatric psychiatry (Davies and O'Mahony 2015; Sys et al. 2020). In our study, benzodiazepines—more specifically lorazepam > 2 mg/day and oxazepam as the most frequently prescribed PRISCUS-PIM and FORTA class D drug, respectively—significantly contributed to PIM prescriptions. Given the increased risk of falls, cognitive side effects, delirogenic potential, and risk of developing dependence, benzodiazepines should be used with caution in elderly psychiatric patients (Davies and O'Mahony 2015; Hefner et al. 2021). Analogous considerations apply to Z-drugs such as zopiclone. Oxazepam is the drug of choice for the treatment of alcohol withdrawal symptoms in many hospitals and is also regularly used in geriatric patients (Kraemer et al. 1999). The use of benzodiazepines can hardly be avoided altogether in this patient population; however, short-to-medium-acting substances such as lorazepam or oxazepam should be preferred to longer acting agents such as diazepam in the treatment of withdrawal symptoms (de Millas et al. 2010; Kraemer et al. 1999). Clomethiazole does not seem to be a suitable alternative for withdrawal treatment in geriatric patients because of its well-known risks of respiratory depression, hypotension, and increased bronchial secretion (de Millas et al. 2010).

The PRISCUS list recommends the use of low-potency antipsychotics such as pipamperone as well as the sleep-inducing antidepressants trazodone and mirtazapine as alternatives to benzodiazepines or Z-drugs for anxiolysis, sedation, and agitation in the context of dementia (Siebert et al. 2013). By contrast, mirtazapine and pipamperone are considered as class C drugs, and trazodone is even labeled as a class D drug according to the FORTA classification (Pazan et al. 2020). Pipamperone is an essential component of delirium therapy and, accordingly, it is frequently used in geriatric psychiatry (Boettger et al. 2017). Although pipamperone is associated with the risk of QT_c_ interval prolongation and seizures, it is preferable to other low-potency antipsychotics such as promethazine because of its lower risk of extrapyramidal motor disturbances and negligible anticholinergic side effects (Kloosterboer et al. 2020).

Mirtazapine also does not exert clinically relevant anticholinergic side effects and derives its sleep-inducing potential from its pronounced antihistaminergic effect (Rothschild-Fuentes et al. 2013). The sedation that frequently occurs under treatment with mirtazapine is usually therapeutically desired, and weight gain is much less pronounced in elderly as compared to younger patients. Trazodone also does not display clinically relevant anticholinergic properties; its sedative effect can rather be explained by the blockade of presynaptic alpha-adrenergic receptors. In addition, trazodone exerts serotonergic effects, which explains its mood-enhancing benefits (Khouzam 2017; Sys et al. 2020). Mirtazapine and trazodone have a significantly better benefit–risk profile in geriatric patients as compared to other antidepressants such as amitriptyline and should therefore be preferred not only for anxiolysis and sedation but also for the treatment of depression in gerontopsychiatric patients. Mirtazapine and trazodone are also used off-label for the treatment of agitation in dementia (Banerjee et al. 2021).

In conclusion, the FORTA classification focuses on isolated adverse effects of substances such as pipamperone, mirtazapine, or trazodone, but does not sufficiently consider their value as substances with comparatively fewer side effects in geriatric psychiatric patients.

The second medication class that accounted for a substantial proportion of PIM prescriptions in our study population were antipsychotics. Remarkably, aripiprazole was the second most frequently prescribed FORTA class D drug in our study population. Yet, aripiprazole does have certain advantages in elderly patients, for example comparatively low risks of QT_c_ interval prolongation or extrapyramidal motor disturbances and only mild anticholinergic side effects (Kirino 2015; Pahwa et al. 2021). Notwithstanding, a Dear Doctor Letter in 2005 pointed out the higher risk of cerebrovascular events with aripiprazole therapy in patients with behavioral disturbances in the context of dementia, so the use of this agent in elderly patients should certainly be viewed critically (Wang et al. 2007).

Olanzapine and clozapine were among the most frequently prescribed PIMs according to the PRISCUS list. Even though both substances only display a low risk of extrapyramidal motor disorders, both have pronounced anticholinergic properties and strong sedating effects (Gareri et al. 2008; Pahwa et al. 2021). In the case of clozapine, other relevant side effects such as the risks of agranulocytosis and myocarditis should also be noted (Mukku et al. 2018). Therefore, the use of olanzapine and clozapine in geriatric psychiatry may be considered with special care, especially in the context of behavioral disorders or psychoses in dementia. By contrast, discontinuation of olanzapine or clozapine in patients with underlying schizophrenia who have been treated with these agents for many years poses the risk of psychotic decompensation and cholinergic rebound, particularly in the case of clozapine, and should thus only be conducted after a careful benefit–risk assessment (Mukku et al. 2018).

Risperidone is rated as a FORTA class C drug (Pazan et al. 2022), whereas it is considered as a suitable therapeutic alternative to other antipsychotics according to the PRISCUS list (Siebert et al. 2013). Although risperidone has the highest potential for extrapyramidal motor side effects among second-generation antipsychotics, its anticholinergic potential is significantly lower compared with, for example, olanzapine (Pahwa et al. 2021). Risperidone is a particularly relevant agent in geriatric psychiatry because it is the only antipsychotic with a proven additional benefit and thus an approval for behavioral disorders and psychoses in dementia (Mühlbauer et al. 2021). Risperidone is also a proven component of delirium therapy (Bocatto et al. 2016). Another alternative in the future may become pimavanserin, which was approved in the United States for the treatment of psychosis in PD and which also appeared to have beneficial effects in behavioral disorders in dementia in some studies (Pahwa et al. 2021).

In summary, our study showed that in the geriatric psychiatry setting, a considerable proportion of patients receive PIMs, but the corresponding number of PIM prescriptions represents only a small proportion of total prescriptions. The use of PIM classification systems to assess drugs with respect to their suitability for elderly patients appears reasonable to improve medication safety in this patient population. One problem with such classifications is that they are not specifically adapted to psychiatric settings. In particular, the FORTA classification is often based merely on the side effect profile and does not specify alternatives, so that its use in geriatric psychiatry may lead to an overestimation of the number of PIM prescriptions. Our study demonstrated that the use of some drugs such as oxazepam, trazodone, or risperidone is evaluated contradictorily by the PRISCUS list and FORTA classification, which can lead to significant differences in the evaluation of medications.

We consider interdisciplinary medication reviews as conducted in our study as a suitable instrument to increase medication safety. In our opinion, this can also serve as an explanation for the relatively low proportion of PIM prescriptions in relation to total prescriptions in our study. Although medication reviews have been demonstrated to be clinically useful, there are no uniform recommendations about which aspects of pharmacotherapy should be discussed during medication reviews (Anderson et al. 2014; Zwietering et al. 2019). Limitations of our study are the monocentric design and the limited number of enrolled patients. Furthermore, the study was conducted in a highly specialized ward of a university hospital; therefore, our results may not be directly transferable to other care structures. Besides, our study does not allow to draw causal inferences between PIM prescriptions and the actual occurrence of ADRs. Future randomized controlled studies should prospectively evaluate if the reduction of PIMs can actually prevent the occurrence of ADRs in elderly psychiatric inpatients.

## Supplementary Information

Below is the link to the electronic supplementary material.

Supplementary file1 (DOCX 33 kb)

## Data Availability

The data that support the findings of this study are available upon reasonable request from the corresponding author.

## Abbreviations

ADR: Adverse drug reaction
FORTA: Fit fOR The Aged
ICD-10: International Statistical Classification of Diseases and Related Health Problems 10th Revision
IQR: Interquartile range
PD: Parkinson’s disease
PIM: Potentially inappropriate medication (for older people)
SD: Standard deviation
