# Supplementary material for: Potentially inappropriate medications according to PRISCUS list and FORTA (Fit fOR The Aged) classification in geriatric psychiatry: a cross-sectional study
Source: J Neural Transm (Vienna). 2022 Sep 2;129(11):1367–75. doi: 10.1007/s00702-022-02541-1 (PMC9550757; doi:10.1007/s00702-022-02541-1)
Supplement: Supplementary file 1 — Supplementary file1 (DOCX 33 kb) [file 702_2022_2541_MOESM1_ESM.docx]

**SUPPLEMENTARY TABLE 1** Absolute and relative frequencies of drugs prescribed in the study population (in alphabetical order)

| **Drug** | **n** | **%** | |
| --- | --- | --- | --- |
| **All drugs** | **2,363** | **100** | |
| Acetylsalicylic acid | 53 | 2.24 |  |
| Agomelatine | 5 | 0.21 |  |
| Allopurinol | 6 | 0.25 |  |
| Alprazolam | 6 | 0.25 |  |
| Amisulpride | 22 | 0.93 |  |
| Amlodipine | 60 | 2.54 |  |
| Amoxicillin | 2 | 0.08 |  |
| Amphotericin B | 2 | 0.08 |  |
| Ampicillin | 1 | 0.04 |  |
| Apixaban | 24 | 1.02 |  |
| Aripiprazole | 12 | 0.51 |  |
| Atorvastatin | 43 | 1.82 |  |
| Atropine | 1 | 0.04 |  |
| Benserazide | 11 | 0.47 |  |
| Beta-acetyldigoxin | 3 | 0.13 |  |
| Betahistine | 7 | 0.30 |  |
| Betamethasone | 8 | 0.34 |  |
| Bisoprolol | 75 | 3.17 |  |
| Brimonidine | 2 | 0.08 |  |
| Brinzolamide | 4 | 0.17 |  |
| Budesonide | 5 | 0.21 |  |
| Buprenorphine | 7 | 0.30 |  |
| Bupropion | 13 | 0.55 |  |
| Calcipotriol | 17 | 0.72 |  |
| Calcitriol | 3 | 0.13 |  |
| Calcium | 12 | 0.51 |  |
| Candesartan | 67 | 2.84 |  |
| Carbidopa | 1 | 0.04 |  |
| Carbimazole | 2 | 0.08 |  |
| Cariprazine | 1 | 0.04 |  |
| Carvedilol | 3 | 0.13 |  |
| Cefazolin | 1 | 0.04 |  |
| Cefotaxime | 1 | 0.04 |  |
| Ceftriaxone | 2 | 0.08 |  |
| Cefuroxime | 2 | 0.08 |  |
| Chlortalidone | 4 | 0.17 |  |
| Cholecalciferol | 75 | 3.17 |  |
| Ciclopirox | 4 | 0.17 |  |
| Ciprofloxacin | 1 | 0.04 |  |
| Citalopram | 7 | 0.30 |  |
| Clavulanic acid | 2 | 0.08 |  |
| Clindamycin | 3 | 0.13 |  |
| Clopidogrel | 2 | 0.08 |  |
| Clozapine | 10 | 0.42 |  |
| Cotrimoxazole | 3 | 0.13 |  |
| Cyclosporine | 3 | 0.13 |  |
| Cyproterone acetate | 5 | 0.21 |  |
| Dabigatran | 1 | 0.04 |  |
| Dapagliflozin | 4 | 0.17 |  |
| Dexamethasone | 2 | 0.08 |  |
| Dextromethorphan | 3 | 0.13 |  |
| Diazepam | 6 | 0.25 |  |
| Diclofenac | 7 | 0.30 |  |
| Digitoxin | 3 | 0.13 |  |
| Digoxin | 5 | 0.21 |  |
| Dimetindene | 2 | 0.08 |  |
| Disodium hydrogen phosphate | 1 | 0.04 |  |
| Donepezil | 2 | 0.08 |  |
| Dorzolamide | 2 | 0.08 |  |
| Doxazosin | 5 | 0.21 |  |
| Dronabinol | 8 | 0.34 |  |
| Dulaglutide | 5 | 0.21 |  |
| Duloxetine | 6 | 0.25 |  |
| Edoxaban | 37 | 1.57 |  |
| Empagliflozin | 3 | 0.13 |  |
| Enalapril | 3 | 0.13 |  |
| Enoxaparin | 2 | 0.08 |  |
| Eplerenone | 1 | 0.04 |  |
| Escitalopram | 2 | 0.08 |  |
| Esomeprazole | 11 | 0.47 |  |
| Etoricoxib | 2 | 0.08 |  |
| Fentanyl | 3 | 0.13 |  |
| Ferrous sulfate | 16 | 0.68 |  |
| Finasteride | 2 | 0.08 |  |
| Flucloxacillin | 2 | 0.08 |  |
| Fluoxetine | 4 | 0.17 |  |
| Flupentixol | 1 | 0.04 |  |
| Folic acid | 30 | 1.27 |  |
| Fresubin® | 4 | 0.17 |  |
| Furosemide | 2 | 0.08 |  |
| Gentamicin | 2 | 0.08 |  |
| Glycopyrronium | 10 | 0.42 |  |
| Haloperidol | 4 | 0.17 |  |
| Heparin | 4 | 0.17 |  |
| Human insulin | 15 | 0.63 |  |
| Hyaluronic acid | 2 | 0.08 |  |
| Hydrochlorothiazide | 28 | 1.18 |  |
| Hydromorphone | 2 | 0.08 |  |
| Hydroxycarbamide | 2 | 0.08 |  |
| Hydroxychloroquine | 8 | 0.34 |  |
| Hylo-Vision® SafeDrop® 0.1 % | 3 | 0.13 |  |
| Hypromellose | 1 | 0.04 |  |
| Ibuprofen | 6 | 0.25 |  |
| Indacaterol | 10 | 0.42 |  |
| Insulin glargine | 2 | 0.08 |  |
| Insulin lispro | 6 | 0.25 |  |
| Ipratropium | 1 | 0.04 |  |
| Irbesartan | 2 | 0.08 |  |
| Kreon® | 6 | 0.25 |  |
| Lactulose | 18 | 0.76 |  |
| Lamotrigine | 6 | 0.25 |  |
| Latanoprost | 4 | 0.17 |  |
| Levetiracetam | 8 | 0.34 |  |
| Levodopa | 12 | 0.51 |  |
| Levofloxacin | 1 | 0.04 |  |
| Levothyroxine | 52 | 2.20 |  |
| Lithium | 12 | 0.51 |  |
| Lorazepam | 50 | 2.12 |  |
| Macrogol | 29 | 1.23 |  |
| Magnesium | 1 | 0.04 |  |
| Melperone | 19 | 0.80 |  |
| Meropenem | 1 | 0.04 |  |
| Metamizole | 40 | 1.69 |  |
| Metformin | 35 | 1.48 |  |
| Metoprolol | 25 | 1.06 |  |
| Mirtazapine | 56 | 2.37 |  |
| Morphine | 1 | 0.04 |  |
| Naloxone | 4 | 0.17 |  |
| Nebivolol | 4 | 0.17 |  |
| Nystatin | 2 | 0.08 |  |
| Octenidine | 4 | 0.17 |  |
| Ofloxacin | 1 | 0.04 |  |
| Olanzapine | 25 | 1.06 |  |
| Omeprazole | 9 | 0.38 |  |
| Opipramol | 1 | 0.04 |  |
| Oxazepam | 21 | 0.89 |  |
| Oxycodone | 2 | 0.08 |  |
| Paliperidone | 6 | 0.25 |  |
| Pantoprazole | 88 | 3.72 |  |
| Paracetamol | 3 | 0.13 |  |
| Paroxetine | 1 | 0.04 |  |
| Phenprocoumon | 2 | 0.08 |  |
| Pipamperone | 67 | 2.84 |  |
| Piperacillin | 1 | 0.04 |  |
| Potassium | 14 | 0.59 |  |
| Potassium dihydrogen phosphate | 1 | 0.04 |  |
| Pravastatin | 3 | 0.13 |  |
| Prednisolone | 19 | 0.80 |  |
| Pregabalin | 12 | 0.51 |  |
| Propranolol | 8 | 0.34 |  |
| Quetiapine | 54 | 2.29 |  |
| Ramipril | 94 | 3.98 |  |
| Rifaximin | 12 | 0.51 |  |
| Risedronic acid | 3 | 0.13 |  |
| Risperidone | 119 | 5.04 |  |
| Rivaroxaban | 16 | 0.68 |  |
| Rosuvastatin | 6 | 0.25 |  |
| Sacubitril | 13 | 0.55 |  |
| Salbutamol | 2 | 0.08 |  |
| Saw palmetto | 1 | 0.04 |  |
| Sertraline | 18 | 0.76 |  |
| Simeticone | 2 | 0.08 |  |
| Simvastatin | 38 | 1.61 |  |
| Sitagliptin | 7 | 0.30 |  |
| Sodium chloride | 8 | 0.34 |  |
| Spironolactone | 36 | 1.52 |  |
| Sterofundin® | 7 | 0.30 |  |
| Stinging nettle | 1 | 0.04 |  |
| Sulbactam | 1 | 0.04 |  |
| Sultamicillin | 2 | 0.08 |  |
| Tamsulosin | 24 | 1.02 |  |
| Tazobactam | 1 | 0.04 |  |
| Thiamine | 20 | 0.85 |  |
| Tianeptine | 3 | 0.13 |  |
| Tilidine | 2 | 0.08 |  |
| Tinzaparin | 90 | 3.81 |  |
| Tiotropium | 2 | 0.08 |  |
| Torasemide | 72 | 3.05 |  |
| Tramadol | 2 | 0.08 |  |
| Trazodone | 12 | 0.51 |  |
| Trospium chloride | 1 | 0.04 |  |
| Valerian | 6 | 0.25 |  |
| Valproic acid | 10 | 0.42 |  |
| Valsartan | 13 | 0.55 |  |
| Venlafaxine | 48 | 2.03 |  |
| Verapamil | 1 | 0.04 |  |
| Vidisic® | 3 | 0.13 |  |
| Vitamin B6 | 4 | 0.17 |  |
| Vitamin B-Komplex® | 20 | 0.85 |  |
| Vitamin C | 2 | 0.08 |  |
| Xipamide | 5 | 0.21 |  |
| Zopiclone | 10 | 0.42 |  |
